# Survey of non-resuscitation fluids administered during septic shock: a multicenter prospective observational study

**DOI:** 10.1186/s13613-019-0607-7

**Published:** 2019-11-27

**Authors:** Anja Lindén-Søndersø, Mårten Jungner, Martin Spångfors, Mohammed Jan, Adam Oscarson, Sally Choi, Thomas Kander, Johan Undén, Donald Griesdale, John Boyd, Peter Bentzer

**Affiliations:** 1 Department of Anesthesiology and Intensive Care, Helsingborg Hospital, Charlotte Yhlens gata 10, 251 87 Helsingborg, Sweden; 2 Department of Intensive and Perioperative Care, Skåne University Hospital, Malmö, Sweden; 3 Department of Clinical Sciences Lund, Anesthesiology and Intensive Care, Kristianstad Hospital, Lund University, Lund, Sweden; 4 Anesthesia Department, College of Medicine, Taibah University, Madinah, Saudi Arabia; 5 Department of Anesthesia and Intensive Care, Skåne University Hospital, Lund, Sweden; 6 Department of Operation and Intensive Care, Hallands Hospital, Halmstad, Sweden; 7 Faculty of Medicine, University of British Columbia, Vancouver, BC Canada; 8 Department of Anesthesiology, Pharmacology & Therapeutics, University of British Columbia, Vancouver, BC Canada; 9 Centre for Heart Lung Innovation, University of British Columbia, Vancouver, BC Canada; 10 Department of Clinical Sciences Lund, Anesthesiology and Intensive Care, Helsingborg Hospital, Lund University, Lund, Sweden

**Keywords:** Septic shock, Non-resuscitation fluids, Fluid balance, Vehicle

## Abstract

**Background:**

The indication, composition and timing of administration of non-resuscitation fluid in septic shock have so far received little attention and accordingly the potential to reduce this source of fluid is unknown. The objective of the study was to quantify and characterize non-resuscitation fluid administered to patients with septic shock.

**Methods:**

This prospective observational study was performed in eight intensive care units in Sweden and Canada during 4 months in 2018. Adult patients with septic shock within 24 h of admission to the intensive care unit were eligible for inclusion. Non-resuscitation fluids were defined as fluids other than colloids, blood products and crystalloids at a rate ≥ 5 ml/kg/h. Indication, volume and type of fluid were recorded during the first 5 days after admission. A maximum of 30 patients could be included per centre. To estimate the potential to reduce administration of non-resuscitation fluid, a pragmatic “restrictive” protocol for administration of non-resuscitation fluids was devised based on the most restrictive practice already in place for non-resuscitation fluids at any of the participating centres. Data are presented as median (interquartile range [IQR]).

**Results:**

A total of 200 patients were included in the study and the 30-day mortality was 35%. Patients received a total of 7870 (4060–12,340) ml of non-resuscitation fluids and 2820 (1430–4580) of resuscitation fluids during the observation period. Median volumes of non-resuscitation and resuscitation fluids were similar at day 1 (1620 [710–2320] and 1590 [520–3000]) ml, respectively) and non-resuscitation fluids represented the largest source of fluid from day 2 and onwards after admission to the ICU. Vehicles for drugs such as vasoactive drugs and antibiotics constituted the largest fraction of non-resuscitation fluids (2400 [1270–4030] ml) during the 5-day observation period. Modelling suggested that volume of non-resuscitation fluids could be reduced by 2840 (1270–4900) ml during the first 5 days of admission to the ICU, mainly through reducing maintenance fluids.

**Conclusions:**

Non-resuscitation fluids constitute the major fraction of fluids administered in the ICU to patients suffering from septic shock and may represent the largest modifiable target to reduce fluid overload.

## Background

Administration of resuscitation fluids intravenously to ensure adequate tissue perfusion is a cornerstone in the treatment of sepsis and septic shock [1]. However, it is increasingly recognized that excessive fluid administration has potentially detrimental side effects such as tissue edema with impaired oxygen delivery and compartment syndromes. Observational studies have suggested that a positive fluid balance is associated with poor outcome [2–4]. These observations have inspired studies which investigated whether conservative resuscitation strategies in patients with septic shock can improve survival or reduce the need for life support and associated complications [5–7].

There are many indications for fluid administration in the critically ill. Observational studies have shown that the majority of fluid administered to patients admitted to an intensive care unit (ICU) is for indications other than maintaining intravascular volume, such as nutrition or as a vehicle for intravenous medications [4, 8, 9]. This is perhaps not a surprising finding in hemodynamically stable patients, but several studies indicate that even in patients with septic shock, a major part of the fluid is given for indications other than volume expansion [6, 10–12]. Interestingly, non-resuscitation fluid appears to be the major source of fluid administered for septic shock already within the first few days of admission to the ICU, meaning that these fluids will contribute to the positive fluid balance often observed in the early phases of septic shock [2, 6].

The indication, composition and timing of administration of non-resuscitation fluid in septic shock have so far received little attention and accordingly the potential to reduce this source of fluid is unknown. Based on these considerations, the objective of the present study was to quantify and characterize the fluid given to patients with septic shock during the first 5 days of admission to the ICU and to assess the potential to reduce the non-resuscitation fluid, by modelling a restrictive fluid protocol. For this purpose, we conducted a prospective observational study in eight intensive care units in Sweden and Canada.

## Methods

### Study design and ethics

We conducted this multicenter observational study on eight different sites: six intensive care units in southern Sweden (Helsingborg, Halmstad, Lund, Kristianstad, Malmö and Varberg), and two in Vancouver, British Columbia, Canada (St Paul’s Hospital and Vancouver General Hospital). Patients were included between March 1st 2018 and June 30th 2018. Follow-up time was 30 days from inclusion. The regional ethical boards in Lund, Sweden (application # 2017/565) and Vancouver, Canada (#H17-03504) approved the study. The ethical board in Sweden required informed consent from patients or their legal surrogates before enrollment, whereas the ethical board in Vancouver waived the need for informed consent. We prepared the manuscript according to the STROBE guidelines for observational studies and registered the study at Clinicaltrials.gov NCT03438097 prior to inclusion of the first patient.

### Patients

We included consecutive adult patients (≥ 18 years) with septic shock per SEPSIS-3 criteria [13] within 24 h of admission to respective ICU. Patients were excluded if they were previously included in the study on a prior admission.

### Data collection

During the first 5 days of admission to the ICU, we registered fluid input, output and fluid balance. Nurses registered fluid input, output and fluid balance manually, in all sites but one. At this site, computer software collected fluid input and fluid balance electronically, while a nurse registered fluid output manually. We collected data on patient characteristics from the electronic medical records/paper records and the electronic records differed across the study sites. A designated researcher at each site transferred the data to the CRF. This researcher was carefully informed on how to register the data onto the CRF and was not blinded to the study objectives.

We specified fluids as either non-resuscitation fluids or resuscitation fluids. The non-resuscitation fluids were subdivided into six groups: vehicle for drugs, enteral and parenteral nutrition, glucose solutions, enteral water and crystalloid administered at a rate of < 5 ml/kg/h [14]. The group ‘vehicle for drugs’ was further subdivided into type of drugs: vasoactive drugs (including inotropes), antibiotics, sedation, analgesics, insulin, potassium, other electrolytes and ‘other drugs’. We defined resuscitation fluids as crystalloids administered at a rate of ≥ 5 ml/kg/h, blood products or colloids and registered glucose solutions primarily given for nutritional purposes, i.e., concentrations of 10–20%. Length of day 1 was calculated from time of admission to the ICU until change of day as defined at the respective site. In cases where ICU admission was less than 5 days, we defined the last day as the time from beginning of the last day until ICU discharge.

Daily fluid balance was determined by subtracting total fluid output, except for perspiration from total fluid intake. Bowel movements were included in the fluid balance according to local protocol. Demographic data were collected from the patient charts. Simplified Acute Physiology Score (SAPS-3) and Sequential Organ Failure Assessment (SOFA) were calculated on the day of admission. Mechanical ventilation or renal replacement therapy (RRT) during any of the first 5 days was registered, as well as source of sepsis and surgery as a source of infection. Length of stay in the ICU, ICU mortality and 30-day mortality were also recorded.

### Modelling of a restrictive fluid protocol

In an attempt to estimate the potential to reduce administration of non-resuscitation fluid, we devised a pragmatic “restrictive” protocol for administration of non-resuscitation fluids based on the most restrictive practice already in place for non-resuscitation fluids at any of the participating centres. In this protocol, we assumed the following: no maintenance fluid was given to patients with a positive cumulative fluid balance, no intravenous glucose was given for nutritional purposes, and enteral nutrition was changed to a concentration of 2 kcal/ml in centres using less concentrated formulas.

### Statistics

No sample size calculation was performed. Based on historical admission rates in the smaller participating ICUs during a typical 4-month period, a maximum inclusion of 30 patients per ICU was set in an attempt to balance the cohort. We performed statistical analyses using GraphPad Prism 8.1.1 (GraphPad Software, La Jolla, CA, USA) and R v. 3.5.2 (R Core Team, Vienna, Austria). We did not use imputation for missing data. Data are presented as medians and interquartile ranges.

## Results

### Demographics

We screened a total of 1946 consecutive patients for eligibility during the study period. Of these, 208 patients were diagnosed with septic shock within 24 h of admission. A total of 8 patients were not included (missing fluid charts = 3, no consent *n* = 3, death before consent could be obtained *n* = 2) leaving 200 patients for inclusion in the analysis. Each centre included a median of 24 patients (min–max 16–30). The 30-day mortality in the cohort was 71/200 (35%) and the most common source of sepsis was abdominal infection. Length of ICU stay was 78 (47–169) h. A detailed description of characteristics is presented in Table 1. Fluid data were missing from one patient on day 2 and 3 due to shift of care to palliation, from one patient on day 4 due to transfer to another ICU, and from one patient on day 5 for unknown reasons. Output data were incomplete in one patient due to lack of urinary catheter.

**Table 1:** Patient characteristics

		Missing data
Number of patients	200	NA
Female sex	71 (36)	0
Age, years	69 (59–77)	0
Weight at admission, kg	78 (66–90)	15
SAPS-3	73 (64–82)	30
SOFA score on day 1	10 (8–12)	0
Highest lactate concentration on day 1, mmol/l	3.6 (2.7–5.2)	0
Length of ICU stay, h	78 (47–169)	0
Alive at ICU discharge	158 (79.0)	0
Alive at 30 days	129 (64.5)	0
Renal replacement therapy	48 (24)	0
Mechanical ventilation	135 (67.5)	0
Surgery	58 (29)	0
Source of sepsis		
Abdominal	71 (35.0)	
Respiratory	70 (34.5)	
Soft tissue	24 (11.8)	
Genitourinary	19 (9.4)	
Cardiovascular	3 (1.5)	
Central nervous system	1 (0.5)	
Unknown	15 (7.4)	

Data are presented as median (IQR) or as number (%). Mechanical ventilation and renal replacement therapy at any time during the study observation period. Surgery as a cause of sepsis or for source control

*NA* non-applicable

### Fluid administration

**Fig. 1:**
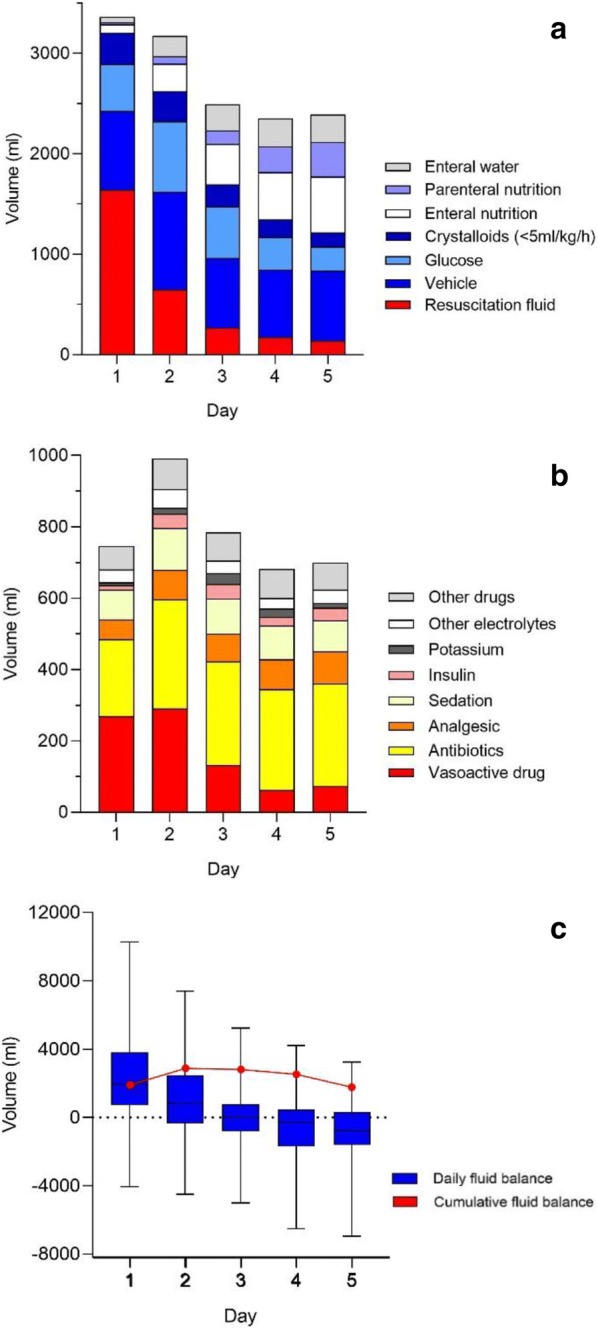
**a** Median daily volume and type of fluids. Data for type of fluids each day are presented as fraction of total daily volume. Please note that sum of the median daily volume does not equal sums of median non-resuscitation and resuscitation fluids over the whole observation period because of the skewed distribution of the data. *N* number of patients. **b** Median daily volume and type of vehicle. Data for type of vehicle each day are presented as fraction of total daily volume. *N* number of patients. **c** Daily and cumulative fluid balance. Daily fluid balance is presented as median, interquartiles and range, and cumulative fluid balance (dots) as median

**Fig. 2:**
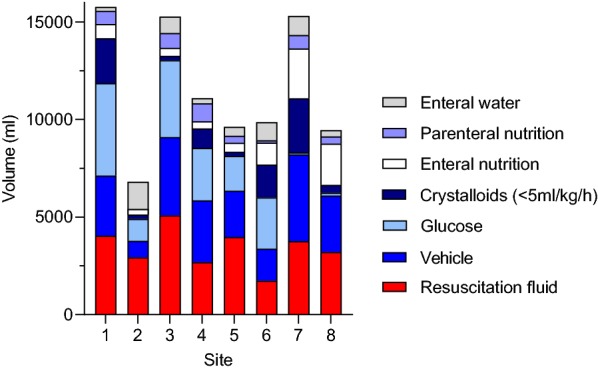
Volume and type of fluids per site day 1–5. Data for type of fluid are presented as fraction of median volume. *N* number of patients

Patients received a median of 7870 (4060–12,340) ml of non-resuscitation fluids and 2820 (1430–4580) ml of resuscitation fluids during the observation period. Daily intake of fluids and daily fluid balance are presented in Fig. 1. Median volumes of non-resuscitation- and resuscitation fluids on day 1 were similar, 1620 (710–2320) and 1590 (530–3000) ml (*p* = 0.072, Wilcoxon rank test), respectively. From day 2 and onwards, daily volume of non-resuscitation fluids was larger than daily volume of resuscitation fluids. A daily breakdown of the different subgroups of non-resuscitation fluids is presented in Fig. 1a and Table 2. Vehicles for drugs constituted the largest fraction of non-resuscitation fluids during the observation period. These vehicles were mainly used for vasoactive drugs and antibiotics as shown in Fig. 1b. Daily fluid balance was positive during the first 3 days in the ICU and cumulative fluid balance was positive during the entire observation period, as demonstrated in Fig. 1c. Total volume of resuscitation and non-resuscitation fluid is presented per site in Fig. 2. There was significant variation in the use of each subgroup of non-resuscitation fluid between the different sites, except for parenteral nutrition (Additional file 1).

**Table 2:** Daily volume of fluids

Day	Resuscitation fluid	Vehicle	Parenteral nutrition	Enteral nutrition	Enteral water	Crystalloids < 5 ml/kg/h	Glucose	Total non-resuscitation fluid
1	1590 (525–3000)	640 (290–1000)	0 (0–0)	0 (0–0)	0 (0–40)	0 (0–590)	210 (0–860)	1620 (710–2320)
2	400 (0–1260)	820 (390–1240)	0 (0–0)	0 (0–350)	0 (0–190)	0 (0–580)	590 (0–1390)	2580 (1560–3450)
3	60 (0–500)	500 (230–1010)	0 (0–0)	130 (0–620)	110 (0–300)	0 (0–410)	210 (0–890)	2220 (1290–2930)
4	0 (0–270)	490 (200–1010)	0 (0–290)	160 (0–670)	120 (0–350)	0 (0–200)	0 (0–480)	2240 (1390–2930)
5	0 (0–200)	570 (310–970)	0 (0–610)	300 (0–800)	160 (0–350)	0 (0–50)	0 (0–290)	2160 (1700–2840)

Volumes are presented as median (IQR). Please note that the sum of the daily medians of the different components of non-resuscitation fluids does not equal the median of the daily total volume of non-resuscitation fluids due to the skewed distribution of the data

### The restrictive fluid protocol

By modelling a restrictive protocol of non-resuscitation fluids in the respective centre, we obtained a theoretical reduction of 2840 (1270–4900) ml per patient of non-resuscitation fluids for the whole cohort, during the observation period (Additional file 2). We observed the largest reductions for glucose solutions in the Swedish centres and for crystalloids as maintenance fluids in nearly half of the Swedish and one of the Canadian centres (Additional file 3).

## Discussion

In patients with septic shock, we demonstrated that the median volume of non-resuscitation and resuscitation fluids was similar at day 1 and that non-resuscitation fluids represented the largest source of fluid from day 2 and onwards after admission to the ICU. Daily fluid balance was positive until day 3 after admission. Fluid as a vehicle for intravenous drugs constituted the major contributor to non-resuscitation fluids.

Resuscitation fluids are administered rapidly to increase preload by an increase in intravascular volume, with crystalloids most commonly administered for this purpose. However, crystalloids may also be administered at a lower rate to provide hydration and to maintain homeostasis, commonly referred to as maintenance fluid. To separate these two indications, we defined a crystalloid as a non-resuscitation fluid if administered at a rate of less than 5 ml/kg/h. This definition aligns with several previous surveys on fluid administration practices [8, 12, 14]. However, it should be noted that some of the studies mentioned below have used other definitions. For instance, crystalloids were defined as a non-resuscitation fluid if administered for reasons other than circulatory impairment [6] or as a resuscitation fluid regardless of infusion rate [7] and in some studies classification of crystalloids was not described in detail [4, 9]. Accordingly, fraction of crystalloids of the non-resuscitation fluids may differ somewhat and highlight the need for a consensus with regard to definitions. Because crystalloids are generally a small fraction of total volume of non-resuscitation fluid, this will not influence the conclusions below.

Previous studies surveying the use of non-resuscitation fluids in patients admitted to ICUs have included a broad set of critically ill patients regardless of their hemodynamic status and need of resuscitation fluids [4, 8, 9]. In this study, we focused our efforts on patients with septic shock, because this is a subgroup of critically ill patients in which the importance of fluid resuscitation is emphasized in guidelines and is also a group where positive fluid balance tends to be a clinical problem. Very little is known about use of non-resuscitation fluids in septic shock, but some data can be extracted from a pilot trial (CLASSIC), comparing two protocols for administration of resuscitation fluids in septic shock [6]. In that study, patients were included approximately 4 h after admission to the ICU and non-resuscitation fluids constituted the major part of the administered fluid already on day 1. Our finding show that this is true also if the early phase of ICU admission, during which resuscitation fluids are most likely to be administered, is included in the day 1 data. The daily fluid balance remained positive up to the 3rd day after admission in our study, in line with previous data from patients with septic shock [6]. With regard to total volume of non-resuscitation fluid during the 5-day observation period, the two studies differ substantially. Patients in the present cohort received approximately 8 l and patients in CLASSIC cohort received approximately 11 l. Differences may be explained by variations in local practice but could also reflect a general change in practice patterns due to increasing awareness of potential adverse effects of intravenous fluids. Another possible explanation is that the median length of stay in the present study was 3 days compared to 6 days in the CLASSIC cohort. Other recent multicenter studies of cohorts of mixed ICU patients reported the total volume of non-resuscitation fluids during the first 3 days of admission to be 5–5.5 l which is similar to our finding of 6.0 l during the same time interval [4, 9]. Similar to our results, these studies also reported that vehicles were the largest fraction of the non-resuscitation fluids.

Based on the growing concern for the adverse effects of excessive fluid administration, several recent pilot studies have assessed if administration of resuscitation fluids can be reduced by “restrictive” protocols or by prediction of fluid responsiveness prior to administration of fluids [5–7, 15]. Data from these studies suggest that the volume of resuscitation fluid can be reduced by 0.8 to 1.2 L during the first 3–5 days of ICU admission. Our results, suggesting that administration of non-resuscitation fluid theoretically could be reduced by a median of 2.8 l during the first 5 days in the ICU, indicate that this approach could potentially have an even larger impact on fluid balance in the ICU.

Several aspects of our modelling could be considered. First, the choice to omit the use of maintenance fluids or glucose could be questioned. However, we are not aware of any studies supporting the use of maintenance fluids in patients that are in a positive fluid balance, nor are we aware of any studies or guidelines suggesting that intravenous glucose should be administered during the acute phase of critical illness [16, 17]. Second, our data suggested that a large part of the vehicles were administered as diluents of antibiotics and vasoactive drugs. To model the potential to reduce vehicles for antibiotics, one could consider both the potential to reduce vehicle volume in already used antibiotics as well as a shift away from antibiotics requiring large volumes of vehicle. Because of the large number of different antibiotics and very limited data on solubility and safety of concentrated antibiotic solutions, such modelling would have been very complex and we therefore elected not to do it. Similarly, we did not model potential reductions in administration of vasoactive drugs, because this group of drugs consists of several different individual drugs with different potentials for concentration. This means that we could have underestimated the potential to reduce the volume of non-resuscitation fluids. Third, the assumption that reductions in volume of non-resuscitation fluid would not be offset by increased administration of resuscitation fluids could be questioned, since crystalloid maintenance fluids will also distribute in the intravascular space. However, intravascular retention of crystalloids over time is most likely very low and is reported to be < 10% in inflammatory conditions meaning that this source of error is reasonably small [18, 19]. Lastly, the physicians caring for the included patients may have aimed for a positive cumulative fluid balance when prescribing non-resuscitation fluids in patients with a perceived preexisting fluid deficit. If so, we may have overestimated the potential to reduce the volume of non-resuscitation fluids somewhat in those patients.

The considerable variation between sites regarding the administration of the different subtypes of non-resuscitation fluids observed in the present study aligns with recently reported data from a multicenter retrospective study in centres in the UK and Canada [4]. This indicates that local practice traditions, rather than evidence-based medicine, play a major role in determining volume as well as the type of non-resuscitation fluid, and highlight the need for more knowledge in this aspect of fluid therapy. Interestingly, none of the sites had any written guidelines with regard to intravenous administration of maintenance fluids or glucose solutions in septic shock. The high variability of current practice has its implications on the design of interventional trials, due to the difficulties in defining a common baseline to which an intervention can be compared. Nevertheless, we believe that our results provide a rationale for an interventional study in which a more restrictive approach of administration of non-resuscitation fluids can be compared to current practice.

While ongoing trials are addressing if restricting resuscitation fluids in septic shock impacts survival [20, 21], an important aspect to bear in mind is that the balance between benefit and harm when reducing resuscitation fluids may be different than the balance when reducing non-resuscitation fluids. Thus, it is important that any intervention with the objective to reduce administration of non-resuscitation fluids should be rigorously assessed in trials regardless of the findings in the ongoing resuscitation fluid trials.

### Strengths and limitations

Strengths of our study include that data are contemporary and collected from multiple sites in both university and regional hospitals. Moreover, the study was also prospectively designed and data were consecutively gathered with high granularity.

Limitations include that even though every effort was made to ensure that fluid input data were captured in a similar way at the different sites, we cannot exclude that smaller amounts of fluids were not registered or that subtle differences in collection of data may have contributed to the inter-site variability seen in the results. Also, we did not standardize registration of bowels movements in the fluid balance which may have resulted in an overestimation of fluid balance in some centres. Other limitations include the small sample size and that only two countries participated in the study. This may limit the validity of our findings in other countries.

## Conclusions

Non-resuscitation fluids constitute the major fraction of fluids administered in the ICU to patients suffering from septic shock and may represent the largest modifiable target to reduce fluid overload.

## Supplementary information

**Additional file 1.** Sources of fluid input day 1–5 by study site. Volumes are presented in millilitres, length of stay presented in hours (median [IQR]). *Kruskal–Wallis test.

**Additional file 2.** Volume and type of fluid during day 1–5 in “standard care” vs “restrictive” protocol. Volumes are presented in millilitres (median [IQR]).

**Additional file 3.** Potential non-resuscitation fluid reduction during day 1–5, per site, in the “restrictive” protocol. Volumes are presented in millilitres (median [IQR]). Please note that the sum of the medians does not equal median of the sum because of the skewed distribution of data.

## Data Availability

The data is available from the corresponding author on reasonable request.

## Abbreviations

ICU: Intensive care unit
IQR: Interquartile range
SAPS III: Simplified Acute Physiology Score, 3rd edition
SOFA: Sequential organ failure assessment
RRT: Renal replacement therapy
