# Supplementary material for: Survey of non-resuscitation fluids administered during septic shock: a multicenter prospective observational study
Source: Ann Intensive Care. 2019 Nov 27;9:132. doi: 10.1186/s13613-019-0607-7 (PMC6881490; doi:10.1186/s13613-019-0607-7)
Supplement: Supplementary file 1 — Additional file 1. Sources of fluid input day 1–5 by study site. Volumes are presented in millilitres, length of stay presented in hours (median [IQR]). *Kruskal–Wallis test. [file 13613_2019_607_MOESM1_ESM.docx]

**Additional file 1. Sources of fluid input day 1-5 by study site.**

| **Site** | **Resuscitation  fluid** | **Vehicle** | **Parenteral nutrition** | **Enteral nutrition** | **Enteral water** | **Crystalloids < 5ml/kg/h** | **Glucose** | **Length of stay** | **No. of patients** |
| --- | --- | --- | --- | --- | --- | --- | --- | --- | --- |
| **All** | 2820 (1430-4580) | 2400 (1270-4030) | 0 (0-0) | 310 (0-1610) | 280 (0-1000) | 600 (0-1990) | 1490 (0-3130) | 79 (47-108) | 200 |
| **1 (SE)** | 2900 (480-6880) | 2610 (1350-4150) | 0 (0-110) | 60 (0-840) | 0 (0-190) | 1000 (230-3130) | 3700 (2150-4730) | 81 (56-110) | 30 |
| **2 (SE)** | 2250 (1500-3980) | 1030 (380-2000) | 0 (0-0) | 0 (0-0) | 1000 (250-2650) | 0 (0-200) | 1180 (290-1660) | 54 (25-86) | 23 |
| **3 (SE)** | 4050 (2950-5700) | 3250 (2000-6550) | 0 (0-1080) | 0 (0-1190) | 740 (0-1770) | 0 (0-0) | 3290 (2430 -4570) | 86 (44-105) | 16 |
| **4 (SE)** | 4210 (2980-5430) | 2600 (1580-4250) | 0 (0-2550) | 0 (0-680) | 100 (0-380) | 740 (0-1700) | 2590 (1010-3130) | 70 (44-110) | 23 |
| **5 (SE)** | 3750 (2420-5990) | 2340 (1470-4440) | 0 (0-830) | 240 (0-680) | 450 (0-710) | 0 (0-0) | 1700 (820-3130) | 75 (30-111) | 25 |
| **6 (SE)** | 1300 (200-2600) | 1250 (770-2560) | 0 (0-0) | 1200 (400-2030) | 630 (150-1510) | 1500 (1000-2020) | 2000 (1570-4050) | 61 (38-102) | 23 |
| **7 (Canada)** | 3600 (1290-5910) | 3680 (2630-5570) | 0 (0-0) | 1600 (80-4580) | 870 (290-1240) | 2320 (1380-3230) | 0 (0-0) | 106 (85-114) | 30 |
| **8 (Canada)** | 3120 (510-4500) | 2320 (1570-3330) | 0 (0-0) | 1360 (250-2900) | 0 (0-750) | 0 (0-190) | 0 (0-0) | 76 (46-107) | 30 |
| **P-value*** | <0.0003 | <0.0001 | 0.0629 | <0.0001 | <0.0001 | <0.0001 | <0.0001 | <0.0085 |  |

Volumes presented in millliters, length of stay presented in hours (median [IQR]). Glucose refers to 5%, 10% and 20% glucose solutions.
*Kruskal-Wallis test
