# Supplementary material for: Survey of non-resuscitation fluids administered during septic shock: a multicenter prospective observational study
Source: Ann Intensive Care. 2019 Nov 27;9:132. doi: 10.1186/s13613-019-0607-7 (PMC6881490; doi:10.1186/s13613-019-0607-7)
Supplement: Supplementary file 2 — Additional file 2. Volume and type of fluid during day 1–5 in “standard care” vs “restrictive” protocol. Volumes are presented in millilitres (median [IQR]). [file 13613_2019_607_MOESM2_ESM.docx]

**Additional file 2. Volume and type of fluid during day 1-5 in “standard care” vs “restrictive” protocol.**

|  | Standard protocol | Restrictive protocol | Change |
| --- | --- | --- | --- |
| Parenteral nutrition | 0 (0-0) | 0 (0-0) | 0 (0-0) |
| Enteral nutrition | 310 (0-1610) | 260 (0-1340) | 0 (0-220) |
| Enteral water | 280 (0-1000) | 280 (0-1000) | 0 (0-0) |
| Crystalloids <5 ml/kg/h | 600 (0-1990) | 0 | 600 (0-1990) |
| Glucose | 1490 (0-3130) | 0 | 1490 (0-3130) |
| Vehicle | 2400 (1270-4030) | 2400 (1270-4030) | 0 (0-0) |
| **Total non-resusc.** | **7870 (4060-12340)** | **4200 (2110-7820)** | **2840(1270-4900)** |

Volumes in millilitres are presented as median (IQR
