# Supplementary material for: Survey of non-resuscitation fluids administered during septic shock: a multicenter prospective observational study
Source: Ann Intensive Care. 2019 Nov 27;9:132. doi: 10.1186/s13613-019-0607-7 (PMC6881490; doi:10.1186/s13613-019-0607-7)
Supplement: Supplementary file 3 — Additional file 3. Potential non-resuscitation fluid reduction during day 1–5, per site, in the “restrictive” protocol. Volumes are presented in millilitres (median [IQR]). Please note that the sum of the medians does not equal median of the sum because of the skewed distribution of data. [file 13613_2019_607_MOESM3_ESM.docx]

**Additional file 3.** **Potential non-resuscitation fluid reduction during day 1-5, per site, in the “restrictive” protocol.**

| **Site** | **Enteral nutrition** | **Crystalloids < 5ml/kg/h** | **Glucose** | **Total** |
| --- | --- | --- | --- | --- |
| **All** | 0 (0-220) | 600 (0-1990) | 1490 (0-3130) | 2840 (1270-4900) |
| **1 (SE)** | 30 (0-420) | 1000 (230-3130) | 3700 (2150-4730) | 5700 (4000-8540) |
| **2 (SE)** | 0 (0-0) | 0 (0-200) | 1180 (290-1660) | 1250 (470-2070) |
| **3 (SE)** | 0 (0-0) | 0 (0-0) | 3290 (2430-4570) | 3770 (2430-5370) |
| **4 (SE)** | 0 (0-0) | 740 (0-1700) | 2590 (1010-3130) | 3330 (1930-4870) |
| **5 (SE)** | 0 (0-0) | 0 (0-0) | 1700 (820-3130) | 2190 (820-3230) |
| **6 (SE)** | 0 (0-0) | 1500 (1000-2020) | 2000 (1570-4050) | 4000 (2570-6140) |
| **7 (Canada)** | 640 (30-1830) | 2320 (1380-3230) | 0 (0-0) | 3790 (2020-4820) |
| **8 (Canada)** | 550 (100-1160) | 0 (0-190) | 0 (0-0) | 950 (160-2340) |
| **P-value*** | <0.0001 | <0.0001 | <0.0001 | <0.0001 |

Volumes in millilitres are presented as median (IQR. Please note that the sum of the medians does not equal
median of the sum because of the skewed distribution of data. *Kruskal-Wallis test
